# Bioethical issues in genome editing by CRISPR-Cas9 technology

**DOI:** 10.3906/biy-1912-52

**Published:** 2020-04-02

**Authors:** Fatma Betül AYANOĞLU, Ayşe Eser ELÇİN, Yaşar Murat ELÇİN

**Affiliations:** 1 Tissue Engineering, Biomaterials and Nanobiotechnology Laboratory, Ankara University Faculty of Science,Ankara University Biotechnology Institute, Ankara University Stem Cell Institute, Ankara Turkey; 2 Biovalda Health Technologies, Inc., Ankara Turkey

**Keywords:** Genome editing, CRISPR-Cas9 technology, bioethical issues, bioethics

## Abstract

Genome editing technologies have led to fundamental changes in genetic science. Among them, CRISPR-Cas9 technology particularly stands out due to its advantages such as easy handling, high accuracy, and low cost. It has made a quick introduction in fields related to humans, animals, and the environment, while raising difficult questions, applications, concerns, and bioethical issues to be discussed. Most concerns stem from the use of CRISPR-Cas9 to genetically alter human germline cells and embryos (called germline genome editing). Germline genome editing leads to serial bioethical issues, such as the occurrence of undesirable changes in the genome, from whom and how informed consent is obtained, and the breeding of the human species (eugenics). However, the bioethical issues that CRISPR-Cas9 technology could cause in the environment, agriculture and livestock should also not be forgotten. In order for CRISPR-Cas9 to be used safely in all areas and to solve potential issues, worldwide legislation should be prepared, taking into account the opinions of both life and social scientists, policy makers, and all other stakeholders of the sectors, and CRISPR-Cas9 applications should be implemented according to such legislations. However, these controls should not restrict scientific freedom. Here, various applications of CRISPR-Cas9 technology, especially in medicine and agriculture, are described and ethical issues related to genome editing using CRISPR-Cas9 technology are discussed. The social and bioethical concerns in relation to human beings, other organisms, and the environment are addressed.

## 1. Introduction

For many years, molecular biologists have sought ways to use cellular repair mechanisms to manipulate DNA through genome editing. In this way, they would have the power to change the genome by correcting a mutation or introducing a new function (Rodriguez, 2016). For this purpose, genome editing technologies were developed (Memi et al., 2018). In recent years, clustered regularly interspaced short palindromic repeats technology (CRISPR-Cas9) has become the most preferred method of gene editing. This technology has advantages such as high accuracy, easy handling, and relatively low cost compared to previous technologies, such as zinc-finger nuclease (ZFN) and transcription activator-like effector nuclease (TALEN). Thanks to these benefits, CRISPR-Cas9 technology can be easily applied in any molecular biology laboratory. 

Genome editing technologies are used in the formation of human disease models in experimental animals and for the understanding of basic gene functions. They also have great therapeutic potential for future treatment of untreated diseases such as certain cancers, genetic disorders, and HIV/AIDS. Today, genome editing in somatic cells is one of the promising areas of therapeutic development (Otieno, 2015). However, various bioethical issues have arisen due to the potential impact of these technologies on the safety of food stocks and clinical applications (Hundleby and Harwood, 2018; Hirch et al., 2019). This review discusses the challenges, possible consequences, and bioethical issues of CRISPR-Cas9 in detail.

## 2. Biology and function of CRISPR-Cas9 technology

Genome editing technologies often work by creating fractures in chromosomal DNA. ZFN, TALEN, and CRISPR-Cas9 are all based solely on nucleases (Kim and Kim, 2014; Roh et al., 2018). The strength of these technologies stems from the ability to create fractures in the desired region of a specific target sequence as determined by the researcher. This allows researchers to modify the genome in practice in any region (Memi et al., 2018).

The creation of changes in the genome depends mainly on the DNA repair capacity of the cells (Lau et al., 2018). All cells have two basic mechanisms for the repair of double chain breaks on DNA. One of them is nonhomologous end joining (NHEJ) and the other is the homologous dependent repair (HDR) mechanism. In NHEJ, the ends of the fractures are quickly connected directly to each other, regardless of the sequence homology, while HDR requires homology to repair the damaged DNA site. In order to achieve homology, the undamaged sister chromatid is used as a template and DNA damage is repaired (Urnov, 2018).

CRISPR-Cas9 is a naturally occurring defense system in prokaryotic organisms that provides resistance to foreign genetic elements such as plasmids and bacteriophages (Barrangau et al., 2007; Marraffini and Sontheimer, 2008). When the virus or plasmid enters a bacterial cell, CRISPR-Cas9 allows the addition of short viral DNA molecules to the CRISPR site. CRISPR sequences (CRISPRs) are short DNA repeats of viral or plasmid origin found in the genomes of bacteria and are defined as clustered regularly interspaced short palindromic repeats. Cas genes (CRISPR-related) are genes that encode nuclease or helicase proteins associated with CRISPR repeat sequences that have the function of cutting or dissolving DNA (Jansen et al., 2002). *Cas9*, a member of the Cas gene family, was isolated from *Streptococcus pyogenes* and is an endonuclease capable of cutting DNA from two active cut regions at both ends of the DNA double helix (Doudna and Charpentier, 2014; Rodriguez, 2016). The CRISPR-Cas system recognizes the DNA of the invading virus or bacterium and directs the Cas protein to destroy foreign DNA (Otieno, 2015).

In the following years, it was discovered that the CRISPR-Cas system can be programmed to find and cut specific target DNA regions, thereby providing genome editing (Jinek et al., 2012; Hsu et al., 2013). As a result of understanding that the human genome can be edited by CRISPR-Cas9, it became clear that genome editing could also be used for therapeutic purposes, and a new era in genetic engineering began (Lau et al., 2018; Roh et al., 2018). 

## 3. Application areas

### 3.1. Animal models 

CRISPR-Cas9 can be used to create animal models to mimic human diseases and to understand disease development by mutating or silencing genes. A mouse model has been developed to determine the harmful effects of mutations in cancer by making mutations that cause the loss of function in tumor suppressor genes or give functions to protooncogenes (Chin, 2015).

Conventional genetically modified (GM) mouse models are produced by gene targeting in embryonic stem cells or transgenesis, which are time-consuming and highly expensive. With CRISPR-Cas9, GM mice can be efficiently produced in a much shorter time (Mei et al., 2016). It can be applied to nonhuman primates such as monkeys. Nonhuman primates are more similar to humans in anatomical, physiological, and genetic terms than rodents (Zhang et al., 2014). Therefore, they are more suitable models than rodents in understanding human biology and disease development (Xin et al., 2016). The first successful application of CRISPR-Cas9 in nonhuman primates, from which a knockout monkey was produced, was realized in 2014 (Niu et al., 2014). However, genome sequences of many nonhuman primates are not yet fully identified. This makes it difficult to design selected single-guide RNAs (sgRNAs) (Gou and Li, 2015; Lou et al., 2016). Therefore, the application of CRISPR-Cas9 in nonhuman primates is still at an early stage.

### 3.2. Genome editing in specific tissues

Researchers have been able to modify the genomes of specific tissues such as liver and brain tissues using hydrodynamic injection and adeno-associated virus (AAV) (Rodriguez et al., 2014; Senis et al., 2014). In a study, CRISPR-Cas9 has been successfully and effectively applied to the mammalian nervous system. A mixture of green fluorescent protein (GFP)-labeled AAV-spCas9 and AAV-spGuide plasmids was transferred in vivo to the hippocampal toothed brain folds of adult male mice (Swiech et al., 2015). It is thought that the number of such applications will grow in the fields of cancer and neuroscience in the following years (Mei et al., 2016).

### 3.3. Multiple gene mutations

CRISPR-Cas9 can be used to generate mutants for target genes. In the first such study by Li et al., six sgRNAs targeting Cas9 mRNA and six different genomic regions encoding the *Tet1*, *Tet2*, and *Tet3* genes were transferred to the cytoplasm of rat embryos (Li et al., 2013). Findings showed that all three Tet genes carried the desired mutations in 59% of newborn rat pups. Successful results were also reported in studies with zebrafish embryos and *Arabidopsis* as well (Ota et al., 2014; Wang et al., 2015).

### 3.4. Epigenome studies

Epigenome studies can be performed in two different ways: genome or epigenome editing (Chen et al., 2014; Huisman et al., 2015). In genome editing, nuclease is used to modify the DNA sequence, whereas in epigenome editing, an effector domain is used and the DNA sequence is not changed. This function is achieved by catalytic inactivation of the Cas9-associated effector domain by replacing the Cas9 protein. Altered effector proteins are used to activate or suppress transcription (Lau and Davie, 2016). In epigenome editing, the epigenome can be modified by changing the proteins that maintain and protect the epigenome. Suppression of DNA methylation as a result of degradation of catalytic domains that accelerate the loss of spherical DNA methylation in human cells and lead to cell death is a good example of epigenome studies using CRISPR-Cas9 (Liao et al., 2015).

Another application is the editing of long nonencoded RNAs (lncRNAs) and enhancer RNAs (eRNAs) that can control gene expression and epigenome processes. In a study, eRNA-expression factors and IncRNA-expression enhancers were suppressed by stimulating deletion mutations in a lymphoma cell line using CRISPR-Cas9 (Pefanis et al., 2015). It is predicted that CRISPR-Cas9 may allow different levels of epigenome modification and facilitate further changes to humans (Liao et al., 2015; Mei et al., 2016).

### 3.5. Treatment of diseases

CRISPR-Cas9 can be applied to cells in vivo or ex vivo. In the in vivo approach, CRISPR-Cas9 is directly transferred to cells in the body using either viral or nonviral methods. In the ex vivo approach, first the cells are removed from the body; then CRISPR is applied to the cells and they are transferred back to the body (Roh et al., 2018). This approach has great potential to develop tissue-based therapies (Rath et al., 2015). Using CRISPR-Cas9, the mutation in the dystrophin protein responsible for the most common form of Duchenne muscular dystrophy was successfully removed (Amoasii et al., 2018; Duchêne et al., 2018; Koo et al., 2018; Long et al., 2018). There are studies to prevent and treat AIDS by inhibiting the entry of HIV into the cell or by removing the HIV genome integrated into the host genome using CRISPR-Cas9 (Saayman et al., 2015). Induced pluripotent stem cells (iPSCs) were successfully produced from cystic fibrosis patients with confirmed F508 deletion in the cystic fibrosis transmembrane regulator (*CFTR*) gene by CRISPR-Cas9 (Firth et al., 2015). There are also studies for cataracts (Wu et al., 2015; Yang et al., 2016) and Parkinson’s disease (Yang et al., 2016). However, recent studies have shown that CRISPR-Cas9 activates the type 1 interferon (INF) pathway, causing a type 1 INF-mediated immune response (Kim et al., 2018; Charleswort et al., 2019). These findings currently limit the use of CRISPR-Cas9 in treatment.

### 3.6. Industrial uses

CRISPR was first used for commercial purposes to make bacterial cultures used in cheese and yogurt production resistant to viral infections (van Erp et al., 2015). One of the applications in agriculture is to produce GM crops (Hundleby and Harwood, 2019). There are attempts to increase the yield in the livestock industry (van Erp et al., 2015). It can be used to control invasive pest species to reverse pesticide and herbicide resistance in insects and weeds or to prevent disease spread (Esvelt et al., 2014). Researchers have succeeded in preventing the spread of genes protecting mosquitoes from harmful malaria parasites (Gantz et al., 2015) and making female mosquitoes infertile in the laboratory (Hammond et al., 2016). Vaccine development is another significant area of interest. The smallpox virus vector (VACV) is used in the eradication and vaccination of smallpox. Using CRISPR-Cas9, the efficiency of marker-free VACV vectors has been increased (Yuan et al., 2015). Another example is the hepatitis B vaccine. In order to prevent viral gene expression and replication, specific regions of the hepatitis B genome were targeted and cut by CRISPR-Cas9 (Ramanan et al., 2015).

### 3.7. RNA editing

Single-stranded RNA (ssRNA) sequences can also be edited by CRISPR-Cas9. In RNA editing, CRISPR-Cas9 consists of a DNA oligonucleotide presenting the PAM (protospacer adjacent motif) region (PAMmer), ssRNA, guide RNA (gRNA), and Cas9 protein. PAMmer acts as a PAM region specifically recognized by Cas9 and directs Cas9 to bind and cut the target ssRNA. 5’-Elongated PAMmers containing bases paired with different ssRNAs and immediately in front of PAM are required for specific binding of target ssRNAs. Since RNA molecules have different functions than DNA, CRISPR-Cas9 can offer a much more flexible application than other genome editing methods (Mei et al., 2016).

### 3.8. Military applications

One of the lesser-discussed application areas of CRISPR-Cas9 technology is its use for military purposes. As is known, a substantial portion of genome editing studies are supported by the defense ministries of the countries. These studies are commonly focused on increasing the tolerance of soldiers against biological or chemical warfare. This technology has the potential to influence human performance optimization (Greene and Master, 2018). Studies are usually concentrated on discovering different genes that can be harnessed from other species (Gracheva et al., 2010) and identifying new genes that can be associated with posttraumatic stress disorder, which is frequently experienced by soldiers (Cornelis et al., 2010). In a study by Zou et al. (2015), researchers developed dog embryos with higher muscle mass using CRISPR-Cas9. Another interesting study showed that the *CMG2* gene, known to cause low sensitivity to anthrax toxin when expressed in small amounts, could be silenced by this technology (Arévalo et al., 2014). However, it should be noted that far more research needs to be conducted for using CRISPR technology in humans as a defense tool against biological and chemical weapons (Greene and Master, 2018).

### 3.9. DNA replacement in human embryos (germline genome therapy)

The most controversial usage of CRISPR-Cas9 is the modification of human embryo DNA, or, in other words, its use for germline genome therapy. In 2015, a group of Chinese researchers led by Junjiu Huang applied CRISPR-Cas9 to remove a mutation that causes β-thalassemia, which is a fatal blood disease, from the human β-globulin (*HBB*) gene in the germline of human embryos. In this research, six abnormal embryos not suitable for in vitro fertilization were used. The mutation could be corrected in only one of the embryos. Although the mutation could be corrected in two other embryos, nontarget effects occurred in other genes. In the other three embryos, the mutation could not be corrected. It has been reported that this technique is not ready for clinical use because of nontarget effects on different genes (Roh et al., 2018; Carroll, 2019). Modifications that occur in germline cells can be transferred to future generations. Scientists think that they can extract genes that cause diseases in the population using CRISPR-Cas9 (Cai et al., 2018; Memi et al., 2018). 

## 4. Bioethical issues

The fact that CRISPR-Cas9 is among the important discoveries of the 21st century is widely accepted in the scientific community and related industries. However, the rapid rise of CRISPR-Cas9 has led to new bioethical, social, and legal issues in medicine, agriculture, livestock, and the environment. Possible risks and bioethical issues related to CRISPR-Cas9 are summarized in the Table. 

**Table:** Possible risks and bioethical issues related to CRISPR-Cas9 technology.

Organism	Risks	Bioethical issues	References
Bacteria	Nontarget mutationsGene drifts	Ecological imbalance	Rodriguez, 2016 Hundleby and Harwood, 2019 Esvelt et al., 2014
Plants	Nontarget mutationsGene drifts	Ecological imbalancePatenting	Shinwari et al., 2017 Hundleby and Harwood, 2019
Animals /chimeric animals	Nontarget mutations	Ecological imbalance Patenting Animal welfare and dignity Threatening of human dignity and identity	Rodriguez, 2016 Polcz and Lewis, 2016 Rodriguez, 2017 Eriksson et al., 2018 Koplin, 2019 Degrazia, 2019 de Graeff et al., 2019
Humans	Nontarget mutations Side effects Cost Genetic mosaicism	Eugenics Informed consent Enhancement Accessibility Patenting Safety Incomplete or over legislations	Otieno, 2015 Rodriguez, 2016 Duardo-Sánchez, 2017 Shinwari et al., 2017 Greene and Master, 2018 Sherkow, 2018 Cathomen et al., 2019 Hirsch et al., 2019

### 4.1. Ecological imbalance

In studies using RNA-targeted gene editing methods based on CRISPR-Cas9, nontarget effects should be examined in depth. Since gene drift will persist in a population, possible off-target mutations will continue in each generation. In addition, the number and effect of mutations may increase as generations progress (Rodriguez, 2016; Hundleby and Harwood, 2019). Another concern is the possibility that genes can be transferred to other species in the environment. Transferring the regulated sequences to other species may result in the transmission of negative characteristics to the associated organisms (Esvelt et al., 2014). The distribution of the properties of the entrained genes among the populations can make control very difficult.

### 4.2. Regulations for consumers

The use of CRISPR-Cas9 to obtain the desired genetic modifications makes it very difficult to identify and regulate genetically modified organisms (GMOs) in the market after they leave the laboratory. Therefore, regulatory agencies, such as the US Food and Drug Administration (FDA), European Medicines Agency (EMA), and others, should consider whether any GMOs are suitable for consumers. However, it is not known exactly how to evaluate the possibilities of a growing market with CRISPR-Cas9 (Ledford, 2015; Hundleby and Harwood, 2019).

One of the dilemmas of CRISPR-Cas9 that concerns all the humanity is patenting. As is known, transgenic organisms of industrial use and also some human gene sequences for clinical purposes have been patented (Rodriguez, 2016; Sherkow, 2018). As technologies such as CRISPR continue to evolve, patent-related issues in many areas of biotechnology will continue to increase in the upcoming years. Even today, there are many such cases of patenting. The best-known case is the patent right case between Zhang and Doudna and Charpentier for the therapeutic use of CRISPR-Cas9 in human cells. In the case concluded on 2 December 2016, it was decided to grant the patent to Caribou Biosciences, which Doudna was the founder of (Donohoue et al., 2018).

### 4.3. Genome editing for enhancement

The editing of human germline cells with CRISPR-Cas9, which will be discussed later in more detail, is prohibited for various safety reasons. However, the rate of application of CRISPR-Cas9 to somatic cells is gradually increasing in order to transfer the desired characteristics to our lives. Many phenotypic characteristics have a genetic component independent of the environment. By utilizing this feature, CRISPR-Cas9 can be used to improve the performance of athletes, to prevent violent behavior, or to reduce dependence (Rodriguez, 2016). Although gene therapy is often used to treat patients for their own benefit, the criminal justice system may require repeater or dangerous offenders to correct the genes associated with violence by genome editing technologies in the future. One of the biggest dilemmas here is to obtain informed consent for an underage person if the intervention is made during the development of the zygote. This will give parents or guardians the right to make decisions on behalf of minors for nonhealth reasons. Furthermore, when socially assessed, some genetically improved populations or individuals may have some advantages in comparison to others in terms of various features such as mental and physical capacity (Brokowski, 2018). Therefore, the use of CRISPR-Cas9 in genome enhancement should be seriously discussed both socially and morally.

### 4.4. Military research

The use of CRISPR technology for military purposes is generally considered within the scope of nontherapeutic enhancement and is covered similarly. From this point of view, related bioethical issues are commonly discussed in terms of concepts of benefit/risk, informed consent, and accessibility (Greene and Master, 2018). A notable bioethical problem is the off-target mutations that have been mentioned in relation to other topics. Off-target mutations can cause many undesirable changes in the genome or even lead to fatal diseases. Current information obtained from studies on off-target mutations caused by CRISPR on the genome is very limited. Therefore, the benefit/risk relationship needs to be evaluated carefully. In addition, the possibility that this technology can be used for the production of new biological weapons is frightening.

Another ambiguous issue that needs to be discussed in military enhancement applications is informed consent. It could be difficult to obtain informed consent forms independently without any interaction among individuals due to military training methods, strict norms, and chains of command. Additionally, some soldiers may have difficulty in understanding the concepts of gene therapy and genome editing, as well as the potential risks and benefits of the applications (Greene and Master, 2018).

One important ethical issue is that the use of such technologies will support ongoing inequalities among military parties (Amoroso and Wenger, 2003). CRISPR is currently an expensive technology. Some developed countries might think of using this technology to further strengthen their defenses and even attack underdeveloped or developing countries. This situation could cause a constant tension, making it difficult to provide an environment of peace and stability worldwide.

### 4.5. Generation of chimeric animals for organ transplantation

Organ transplantation is the replacement of an organ that cannot function in an individual’s body with a healthy organ from a living donor or cadaver. The primary purpose is to save the life of the patient, who is in danger of organ failure, and to increase the lifespan and quality of life (Black et al., 2018). The development of chimeric animals may prevent patients from spending precious time waiting for an appropriate donor.

Bioethical issues in the generation of chimeric animals arise from the fact that chimeras contain human nerve and germ cells (Polcz and Lewis, 2016). The two main issues can be summarized as defining the order of nature and the moral disorders caused by how the organism is treated depending on whether the organism is accepted as human or animal. Some people think that chimeric embryos will affect human dignity and identity because they have the power to develop organisms with human-derived cells and tissues. The others state that chimeric organisms containing human cells cannot turn into humans and therefore will not affect human dignity. They also argue that the human-like features imparted to chimeras will neither affect the biological environment nor the moral status of animals and will never reach human consciousness (Koplin, 2019; Degrazia, 2019).

### 4.6. Animal welfare and dignity

Animal welfare is another bioethical concern encountered during the application of genome editing technologies on animals. First of all, the possibility of off-target mutations in the genome can lead to diseases or different side effects in animals (Ishii, 2017a; Schultz-Bergin, 2018; de Graeff et al., 2019). Such a situation will adversely affect animal welfare (Rodriguez, 2017).

The second bioethical issue to be discussed could be the concerns about “animal dignity” (Eriksson et al., 2018) and alterations in their natural environments and physiological needs (Manesh et al., 2014). Some studies have stated that the use of animals as objects only serving for humans is not ethically or morally acceptable (Martinelli et al., 2014; Fung and Kerridge, 2016; Greenfield, 2017), and such practices can lead to greater control over humans on animals (Ishii, 2017a; de Graeff et al., 2019). Some others think that animals are not bound by any moral law and therefore there is no need for a discussion regarding animal dignity (Heeger, 2015; Shriver and McConnachie, 2018). Schultz-Bergin (2017) stated that animal rights, welfare, and dignity will not be adversely affected since these animals will occur through genome editing technologies. The existence of contrary opinions on this matter indicates that the mentioned bioethical issues will be on the agenda for a long time.

### 4.7. CRISPR-Cas9 for human germline 

The potential for using CRISPR-Cas9 for genome editing in the human germline has raised serious ethical debates. Until 2015, all therapeutic applications in humans were performed in somatic cells using genome editing technologies. However, in 2015, the editing of the human germline performed by Chinese scientist Huang and his team with CRISPR-Cas9 raised new social, moral, and bioethical issues (Liang et al., 2015; Ormond et al., 2017). Bioethical issues caused by genome editing in the germline can be classified into two main topics depending on the success and failure of genome editing technologies (Ormond et al., 2017; Greely, 2019).

#### 4.7.1. Issues that may occur in the failure of germline genome editing

Some of the ethical dilemmas of genome editing in the germline arise from the fact that changes in the genome can be transferred to the next generations. Therapeutic genome editing in somatic cells generally does not cause significant concerns when assessing the risk/benefit balance and the use of informed consent. The application of CRISPR-Cas9 in the germline is considered more problematic because of the risk of causing various mutations and side effects and transferring undesirable changes to future generations (Cyranoski and Reardon, 2015; Brokowski, 2018; Cai et al., 2018; Halpern et al., 2019). In fact, Huang and his team found that nontarget mutations in the genome occurred and the study was terminated earlier than planned (Liang et al., 2015). Nontarget mutations are unintentional mutations in the genome and may have harmful effects on the organism as these mutations can lead to cell death or transformation (Zhang et al., 2014). Frighteningly, researchers have found that mutations caused by CRISPR-Cas9 in embryos are much more common than in mouse or human adult cells (Cyranoski and Reardon, 2015). In a study performed with human embryos, it was stated that nontarget mutations occur only in the exon regions and therefore the number of mutations may be much higher than expected (Liang et al., 2015). Due to the high risk of nontarget mutations, some scientists argue that genome editing studies in germline cells should be terminated and its future should be discussed (Cyranoski and Reardon, 2015). Some scientists state that newly developed CRISPR-Cas9 could reduce or even prevent the number of nontarget mutations. In this method, the efficiency of CRISPR-Cas9 was increased by using Cas9-regulated human iPSCs in region-specific gene targeting (Yumlu et al., 2019).

Another bioethical dilemma is the cost of germline genome editing. Genome editing is an expensive technology (Wilson and Carroll, 2019). While families in rich countries may have the power to cover this cost, families in developing countries may not. This situation may cause children born in developed countries to have an unfair advantage in terms of various characteristics such as intelligence and physical state compared to children in other countries (Otieno, 2015).

CRISPR-Cas9 is based on the use of nuclease enzymes. The nuclease enzymes used may not be as effective as desired and not be able to cut all copies of the target gene, or the cell may begin to divide before genome editing is completed. As a result, a condition called genetic mosaicism can occur (Lanphier et al., 2015). Genetic mosaicism is the presence of genetically different somatic cell populations in an organism and is often masked. Mosaicism can also lead to major phenotypic changes, the formation of fatal genetic mutations (Capalbo and Rienzi, 2017), and some genetic diseases such as Down, Klinefelter, and Turner Syndromes (Otieno, 2015). Therefore, the nuclease cleavage sites should be exactly confirmed and the possibility of mosaicism should be completely eliminated.

One of the important bioethical issues is side effects in embryos. It is pointed out that the possible side effects cannot be predicted before birth and the consequences are not clearly known (Otieno, 2015; Brokowski, 2018). Controls can only be performed in a small group of cells. This limitation causes the effects of genome editing on embryos to be unknown and unprevented until birth. In fact, it should be considered that it may take years for many potential problems to emerge (Lanphier et al., 2015; Halpern et al., 2019).

#### 4.7.2. Issues that may occur in the successful application of germline genome editing

The first of the bioethical issues of successful germline genome editing is the use for nontherapeutic changes (Lanphier et al., 2015; Greely, 2019). Such uses will lead to new questions about breeding (eugenics) of the human species and its position in the universe (Yang, 2015). In one study, the fur color of rats was successfully changed by genome editing (Yoshimi et al., 2014). It is possible that the skin color of people could be changed in the future. Since the characteristics of individuals can be determined by genome editing rather than blood relations, the possibility that children with similar physical and mental health can be born in the same way should be considered (Ishi, 2015).

The second bioethical issue is what the fate of children born using genome editing will be. From whom or where informed consent will be obtained in the case of undesirable effects on behalf of genome-edited children and whether informed consent will give detailed information are important questions (Beriain and del Cano, 2018; Neuhaus and Zacharias, 2018; Sykora, 2018; Knoppers and Kleiderman, 2019). While clear informed consent can be given for genome-edited somatic cells to be used in clinical trials, it is an enigma to whom and how to give precise information about the potential risks involved in germline editing (Lanphier et al., 2015; Neuhaus and Zacharias, 2018; Knoppers and Kleiderman, 2019).

In December 2015, the International Summit on Human Gene Editing was convened to discuss the social, moral, and bioethical issues caused by genome editing in the human germline. The results of the summit concluded that basic and clinical investigations should be continued in accordance with the appropriate legal and ethical regulations; however, genome editing on gametocytes and embryos that would cause hereditary changes in humans was found to be irresponsible. It was therefore emphasized that the use of CRISPR-Cas9 on the human germline should be postponed until a solution is found for existing bioethical, social, legal, and technical concerns and issues (Baltimore et al., 2015). In addition, it was agreed to establish an international forum where such concerns could be addressed continuously and the studies in different countries could be organized together (Baltimore et al., 2015; Lanphier et al., 2015; Olson, 2015). The NIH announced that genome editing studies in human embryos will not be financially supported (Collins, 2015). In spite of the joint decisions that were made, in February 2016, British scientists were allowed to use CRISPR-Cas9 and similar technologies in human embryos for research purposes only (https://www.bbc.com/news/health-35459054). In March 2017, the US National Academy of Sciences and the American Society of Human Genetics published a position statement stating that they should be aware of the scientific and bioethical issues that can be caused by germline genome editing, but, on the other hand, research should continue (Ormond et al., 2017). As of January 2020, 24 countries have forbidden genome editing in human embryos by law and 9 countries have banned it by guidelines. However, there are countries that do not impose strict prohibitions on germline genome editing (Ishii, 2017b; Lau et al., 2018; Macintosh, 2019).

## 5. Discussion and future directions

Thanks to its high accuracy, ease of use, and relatively low cost, CRISPR-Cas9 offers a wide range of applications for many people in the medical, agricultural, livestock, and environmental sectors. Furthermore, its precision and accuracy are much higher compared to older technologies such as ZFN and TALEN (Mittal, 2019). The powerful effects of CRISPR-Cas9 have raised many social, moral, and bioethical issues. 

Discussions have generally focused on the social, bioethical, and legal consequences of using genome editing technology in human germline cells. Scientists generally agree that CRISPR-Cas9 should be allowed for use in the creation of human disease models, and in understanding the development and molecular mechanisms of diseases; however, it should be prohibited for the purposes of eugenics or enhancement. When ethical issues, safety concerns, and application difficulties are considered together, it is predicted that therapeutic genome editing in human embryos will not be possible in the near future. Thus, the risk of hereditary nontarget genetic mutation is higher than the possible treatment benefits and it affects the principle of intentional harm. Nevertheless, it is clear that scientists will apply CRISPR-Cas9 in germline cells in the future if solutions are found to the issues mentioned here (Duardo-Sánchez, 2017; Hirsch et al., 2019). CRISPR-Cas9 must be fully reliable for therapeutic use in germline cells. Social, legal, and bioethical issues should be discussed in detail once genome editing technologies have reached the permissible level of safety for clinical applications in the prevention of genetic diseases (Rossant, 2018; Cathomen et al., 2019). Subsequently, regulatory laws that may eliminate breaches of germline genome editing will need to be reassessed (Rodriguez, 2016; Duardo-Sánchez, 2017; Cathomen et al., 2019; Macintosh, 2019). The therapeutic use of CRISPR-Cas9 and its rapid rise in the medical field are expected to continue. While studies on the use of CRISPR-Cas9 for clinical purposes are continuing, the necessary legal, social, and ethical legislation should be put into practice as soon as possible and the public conscience should not be ignored.

On the other hand, the potential effects of CRISPR-Cas9 in other areas should not be forgotten. CRISPR-Cas9 is not just about social and bioethical issues related to people. Interactions with other organisms and the environment, such as the consideration of the principle of intentional harm in risk assessment, safety measures to prevent ecological degradation, or potential use in genetic enhancement of animals and agriculture products should also be discussed (Rodriguez, 2016; Hirsch et al., 2019). There are serious concerns about changes in the natural ecosystem that may occur if the GMOs produced with CRISPR-Cas9 are released to the ecosystem in a controlled or uncontrolled manner. Considering the applications of CRISPR-Cas9 that protect mosquitoes from malaria parasites (Gantz et al., 2015) or make female mosquitoes infertile (Hammond et al., 2016), the effect of GM mosquitoes on other organisms with which they are associated in their ecosystems cannot be predicted. It is clear that small-scale research in the laboratory does not fully reflect possible changes in the natural ecosystem (Carroll, 2017). In agriculture, another concern about GMOs produced with CRISPR-Cas9 is whether they will be accepted by the public. GMOs that were produced using different technologies in the past faced harsh public reactions (Carroll, 2017). Furthermore, the fact that GMOs produced with CRISPR-Cas9 are difficult to identify outside the laboratory raises safety concerns (Shinwari et al., 2017). Before the launch of such products, the necessary explanations and declarations should be made by the authorities in a transparent and clear manner in order to prevent misjudgments and questions that may occur in the public, and precautions and arrangements should be established to ensure the safety of the public. 

Another issue to consider about CRISPR-Cas9 is patenting. Patenting can considerably limit the application of such technologies. Unilateral patenting can significantly increase the profitability of biotechnology companies, which may lead to a rise of bioethical issues. There is disagreement in the scientific community regarding the patenting or nonpatenting of GMOs to be used specifically for therapeutic purposes (Shinwari et al., 2017; Sherkow, 2018). However, there are some who think that patenting will help to eliminate and regulate the deficiencies in the field (Rodriguez, 2016; Shinwari et al., 2017). It should not be forgotten that the most important of these debates about patenting is commercialization and the release of only reliable products.

In recent years, deals between the scientific community and the pharmaceutical and biotechnology sectors for the therapeutic use of CRISPR-Cas9 have raised public safety concerns (Shinwari et al., 2017; Carroll, 2019) The guidelines and legislations that will regulate the content and application of these deals should be prepared as quickly as possible and shared with the public. Due to the challenges and bioethical issues of CRISPR-Cas9, the scientific community and other interested bioethical, social, legal, and governmental parties should be provided with a detailed guide for future processing and use of this technology (Otieno, 2015; Shinwari et al., 2017; Cathomen et al., 2019). In this way, a long-term policy can be developed that will support the scientific development of CRISPR-Cas9 technology together with the discussion of the possible problems in advance and preparation of solution plans.

## References

[ref0] (2018). Gene editing restores dystrophin expression in a canine model of Duchenne muscular dystrophy. Science.

[ref1] (2003). The human volunteer in military biomedical research. Military Medical Ethics.

[ref2] (2014). Targeted silencing of anthrax toxin receptors protects against anthrax toxins. Journal of Biological Chemistry.

[ref3] (2015). A prudent path forward for genomic engineering and germline gene modification. Science.

[ref4] (2007). CRISPR provides acquired resistance against viruses in prokaryotes. Science.

[ref5] (2018). Gene editing in human embryos. A comment on the ethical issues involved. The Ethics of Reproductive Genetics. Cham.

[ref6] (2018). Solid organ transplantation in the 21st century. Annals of Translational Medicine.

[ref7] (2018). Do CRISPR germline ethics statements cut it. CRISPR Journal.

[ref8] (2018). The forty years of medical genetics in China. Journal of Genetics and Genomics.

[ref9] (2017). Focus: Genome editing: genome editing: past, present, and future. Yale Journal of Biology and Medicine.

[ref10] (2019). The human genome editing race: loosening regulatory standards for commercial advantage? Trends in Biotechnology 37 (2.

[ref11] (2019). Identification of preexisting adaptive immunity to Cas9 proteins in humans. Nature Medicine.

[ref12] (2013). Induced DNA demethylation by targeting ten-eleven translocation 2 to the human ICAM-1 promoter. Nucleic Acids Research.

[ref13] (2015). CRISPR-Cas9 Therapeutics: A Technology Overview.

[ref14] (2015). Statement on NIH Funding of Research Using Gene-Editing Technologies in Human Embryos.

[ref15] (2010). Genetics of post-traumatic stress disorder: review and recommendations for genome-wide association studies. Current Psychiatry Reports.

[ref16] (2015). Chinese scientists genetically modify human embryos. Nature News.

[ref17] (2019). The ethics of genome editing in non-human animals: a systematic review of reasons reported in the academic literature. Philosophical Transactions of the Royal Society B.

[ref18] (2019). Human-animal chimeras, “human” cognitive capacities, and moral status. Hastings Center Report.

[ref19] (2014). The new frontier of genome engineering with CRISPR-Cas9. Science.

[ref20] (2017). CRISPR-Cas in medicinal chemistry: applications and regulatory concerns. Current Topics in Medicinal Chemistry.

[ref21] (2018). CRISPR-induced deletion with saCas9 restores dystrophin expression in dystrophic models in vitro and in vivo. Molecular Therapy.

[ref22] (2018). Invited review: Breeding and ethical perspectives on genetically modified and genome edited cattle. Journal of Dairy Science.

[ref23] (2014). Concerning RNA-guided gene drives for the alteration of wild populations. Elife.

[ref24] (2015). Functional gene correction for cystic fibrosis in lung epithelial cells generated from patient iPSCs. Cell Reports.

[ref25] (2016). Gene editing advance re-ignites debate on the merits and risks of animal to human transplantation. Internal Medicine Journal.

[ref26] (2015). Highly efficient Cas9-mediated gene drive for population modification of the malaria vector mosquito Anopheles stephensi. Proceedings of the National Academy of Sciences of the USA.

[ref27] (2010). Molecular basis of infrared detection by snakes. Nature.

[ref28] (2019). Human germline genome editing: an assessment. CRISPR Journal.

[ref29] (2018). Ethical issues of using CRISPR technologies for research on military enhancement. Journal of Bioethical Inquiry.

[ref30] (2017). Editing mammalian genomes: ethical considerations. Mammalian Genome.

[ref31] (2015). Targeted genome editing in primate embryos. Cell Research.

[ref32] (2019). Societal and ethical impacts of germline genome editing: How can we secure human rights?. CRISPR Journal.

[ref33] (2016). A CRISPR-Cas9 gene drive system targeting female reproduction in the malaria mosquito vector Anopheles gambiae. Nature Biotechnology.

[ref34] (2015). Dignity only for humans? A controversy. The Cambridge Handbook of Human Dignity: Interdisciplinary Perspectives.

[ref35] (2015). Ethical considerations in chimera research. Development.

[ref36] (2019). Ethics assessment in research proposals adopting CRISPR technology. Biochemia Medica.

[ref37] (2015). Prolonged re-expression of the hypermethylated gene EPB41L3 using artificial transcription factors and epigenetic drugs. Epigenetics.

[ref38] (2017a). Genome-edited livestock: ethics and social acceptance. Animal Frontiers.

[ref39] (2017b). Germ line genome editing in clinics: the approaches, objectives and global society. Briefings in Functional Genomics.

[ref40] (2014). A guide to genome engineering with programmable nucleases. Nature Reviews Genetics.

[ref41] (2018). CRISPR RNAs trigger innate immune responses in human cells. Genome Research.

[ref42] (2019). Heritable genome editing: Who speaks for “future” children?. CRISPR Journal.

[ref43] (2018). Functional rescue of dystrophin deficiency in mice caused by frameshift mutations using campylobacter jejuni Cas9. Molecular Therapy.

[ref44] (2019). Human-animal chimeras: the moral insignificance of uniquely human capacities. Hastings Center Report.

[ref45] (2015). Don’t edit the human germ line. Nature News.

[ref46] (2018). Gene editing of stem cells for kidney disease modelling and therapeutic intervention. Nephrology.

[ref47] (2016). The discovery and development of the CRISPR system in applications in genome manipulation. Biochemistry and Cell Biology.

[ref48] (2015). CRISPR, the disruptor. Nature.

[ref49] (2013). Simultaneous generation and germline transmission of multiple gene mutations in rat using CRISPR-Cas systems. Nature Biotechnology.

[ref50] (2015). CRISPR/Cas9-mediated gene editing in human tripronuclear zygotes. Protein & Cell.

[ref51] (2015). Targeted disruption of DNMT1, DNMT3A and DNMT3B in human embryonic stem cells. Nature Genetics.

[ref52] (2018). Correction of diverse muscular dystrophy mutations in human engineered heart muscle by single-site genome editing. Science Advances.

[ref53] (2019). Heritable genome editing and the downsides of a global moratorium. CRISPR Journal.

[ref54] (2014). Ethical issues of transplanting organs from transgenic animals into human beings. Cell Journal (Yakhteh).

[ref55] (2008). CRISPR interference limits horizontal gene transfer in staphylococci by targeting DNA. Science.

[ref56] (2014). De-extinction: a novel and remarkable case of bio-objectification. Croatian Medical Journal.

[ref57] (2016). Recent progress in CRISPR/Cas9 technology. Journal of Genetics and Genomics.

[ref58] (2018). CRISPR/Cas9 gene-editing: Research technologies, clinical applications and ethical considerations. Seminars in Perinatology.

[ref59] (2019). Gene editing in clinical practice: where are we?. Indian Journal of Clinical Biochemistry.

[ref60] (2018). Compassionate use of gene therapies in pediatrics: an ethical analysis. Seminars in Perinatology.

[ref61] (2014). Generation of gene-modified cynomolgus monkey via Cas9/RNA-mediated gene targeting in one-cell embryos. Cell.

[ref62] (2016). International Summit on Human Gene Editing: A Global Discussion.

[ref63] (2017). Human germline genome editing. American Journal of Human Genetics.

[ref64] (2014). Multiple genome modifications by the CRISPR/Cas9 system in zebrafish. Genes to Cells.

[ref65] (2015). CRISPR-Cas9 human genome editing: challenges, ethical concerns and implications. Journal of Clinical Research and Bioethics.

[ref66] (2014). Regulating gene drives. Science.

[ref67] (2016). CRISPR-Cas9 and the non-germline non-controversy. Journal of Law and the Biosciences.

[ref68] (2015). CRISPR/Cas9 cleavage of viral DNA efficiently suppresses hepatitis B virus. Scientific Reports.

[ref69] (2015). The CRISPR-Cas immune system: biology, mechanisms and applications. Biochimie.

[ref70] (2016). Ethical issues in genome editing using Crispr/Cas9 system. Journal of Clinical Research and Bioethics.

[ref71] (2017). Ethical issues in genome editing for non-human organisms using CRISPR/Cas9 system. Journal of Clinical Research and Bioethics.

[ref72] (2014). AAV-CRISPR: a new therapeutic approach to nucleotide repeat diseases. Molecular Therapy.

[ref73] (2018). CRISPR Craft: DNA editing the reconstructive ladder. Plastic and Reconstructive Surgery.

[ref74] (2018). Gene editing in human development: ethical concerns and practical applications. Development.

[ref75] (2017). The dignity of diminished animals: Species norms and engineering to improve welfare. Ethical Theory and Moral Practice.

[ref76] (2018). Is CRISPR an ethical game changer. Journal of Agricultural and Environmental Ethics.

[ref77] (2014). CRISPR/Cas9-mediated genome engineering: an adeno-associated viral (AAV) vector toolbox. Biotechnology Journal.

[ref78] (2018). The CRISPR patent landscape: past, present, and future. CRISPR Journal.

[ref79] (2017). Ethical issues regarding CRISPR-mediated genome editing. Current Issues in Molecular Biology.

[ref80] (2018). Genetically modifying livestock for improved welfare: a path forward. Journal of Agricultural and Environmental Ethics.

[ref81] (2015). In vivo interrogation of gene function in the mammalian brain using CRISPR-Cas9. Nature Biotechnology.

[ref82] (2018). Germline gene therapy in the era of precise genome editing: how far should we go? In: Soniewicka M (editor. The Ethics of Reproductive Genetics. Cham.

[ref83] (2018). Genome Editing BC (before CRISPR): lasting lessons from the “old testament”. CRISPR Journal.

[ref84] (2015). The history and market impact of CRISPR RNA-guided nucleases. Current Opinion in Virology.

[ref85] (2015). Egg cell-specific promoter-controlled CRISPR/Cas9 efficiently generates homozygous mutants for multiple target genes in Arabidopsis in a single generation. Genome Biology.

[ref86] (2019). The daunting economics of therapeutic genome editing. CRISPR Journal.

[ref87] (2015). Correction of a genetic disease by CRISPR-Cas9-mediated gene editing in mouse spermatogonial stem cells. Cell Research.

[ref88] (2016). Application of the genome editing tool CRISPR/Cas9 in non-human primates. Zoological Research.

[ref89] (2015). Thinking Towards the Future of CRISPR/Cas9.

[ref90] (2016). CRISPR/Cas9: implications for modeling and therapy of neurodegenerative diseases. Frontiers in Molecular Neuroscience.

[ref91] (2014). Allele-specific genome editing and correction of disease-associated phenotypes in rats using the CRISPR–Cas platform. Nature Communications.

[ref92] (2015). A marker-free system for highly efficient construction of vaccinia virus vectors using CRISPR Cas9. Molecular Therapy-Methods & Clinical Development.

[ref93] (2019). Efficient gene editing of human induced pluripotent stem cells using CRISPR/Cas9. CRISPR Gene Editing.

[ref94] (2014). CRISPR/Cas9 for genome editing: progress, implications and challenges. Human Molecular Genetics.

[ref95] (2014). Experimental primates and non-human primate (NHP) models of human diseases in China: current status and progress. Zoological Research.

